# Retrieval of an IVCF retained for over 6 years via femoral venous approach using a large-bore sheath: a case report and literature review

**DOI:** 10.3389/fcvm.2025.1617684

**Published:** 2025-06-24

**Authors:** Gang Yuan, Wei Hu, Weiming Wang, Yanneng Xu, Ran Cui, Xun Zhang, Jianming Luo, Guangyan Si

**Affiliations:** ^1^ Department of Intervention & Vascular, The Affiliated Traditional Chinese Medicine Hospital, Southwest Medical University, Luzhou, China; ^2^ Department of General Surgery (Vascular Surgery), The Affiliated Hospital of Southwest Medical University, Luzhou, China; ^3^ Department of Oncology, The First People’s Hospital of Neijiang, Neijiang, Sichuan, China

**Keywords:** inferior vena cava filter, prolonged retention, retrieval, large-bore vascular sheath, case report

## Abstract

Prolonged retention of inferior vena cava filters (IVCF) predisposes patients to complications, including caval thrombosis, filter tilt, wall adherence, fibrotic adhesion, migration, and perforation, posing significant risks to patient health. Retrieval of long-term retained filters is challenging, as standard retrieval techniques often prove ineffective. Advanced strategies are therefore required to improve success rates. Herein, we report a case of an IVCF retained for over 6 years, in which the patient developed acute thrombosis of the inferior vena cava (IVC) and iliac veins following recent discontinuation of anticoagulation. Concurrently, the retrieval hook was embedded in fibrotic tissue with wall apposition, and the filter struts had perforated the vascular wall with dense adhesions. Initial attempts using a standard retrieval kit failed. Subsequently, a loop snare technique was employed to dissect perihook fibrotic tissue, successfully engaging the retrieval hook. However, due to the filter's firm incorporation into the IVC, the hook straightened under traction, resulting in retrieval failure. Ultimately, the stubborn filter was successfully removed via a retrograde approach using a 20F vascular sheath through the femoral vein. By detailing this case and reviewing relevant literature, we aim to provide insights into advanced retrieval strategies for challenging IVCF, particularly those with prolonged dwell times.

## 1 Introduction

The inferior vena cava filter (IVCF) is a protective device commonly implanted in patients with contraindications to anticoagulation or those at high risk of thromboembolism from lower extremity deep vein thrombosis (DVT) to prevent fatal pulmonary embolism (PE) (1–3). However, prolonged filter dwell time increases the likelihood of filter-related complications, including wall adherence, fibrotic adhesion, caval wall perforation, filter migration, and IVC thrombosis (4, 5). Due to these risks, retrievable filters have become the preferred option in contemporary practice (6). Although retrievable IVCFs demonstrate irreplaceable efficacy in reducing PE incidence, clinical evidence indicates that filters retained beyond 30 days significantly elevate complication rates (7). Therefore, timely retrieval is critical once the thromboembolic risk subsides and anticoagulation is deemed safe (1, 2).

Most IVCF within the recommended retrieval window can be removed using standard snare techniques involving simple snare engagement and sheath withdrawal. However, complex cases, such as prolonged retention causing hook embedment, severe tilt, or endothelialisation, often defy conventional methods, leading to “IVCF retrieval failure” (8). Advanced techniques, including loop snare technique, balloon angioplasty, forceps-assisted extraction (e.g., bronchial or endoscopic forceps), and excimer laser sheath utilisation, are required to improve success rates in such scenarios (9, 10). Despite these innovations, certain refractory filters remain resistant to extraction, necessitating individualised advanced strategies. Herein, we present a case of a 6-year retained IVCF complicated by acute IVC and iliac vein thrombosis post-anticoagulation discontinuation. The retrieval hook exhibited wall apposition with dense fibrotic encapsulation, while the struts demonstrated vascular wall perforation and firm adhesions. Standard snare and loop techniques initially failed due to hook straightening under traction. Ultimately, successful retrieval was achieved via a retrograde femoral venous approach using a 20F vascular sheath combined with snare assistance. By detailing this case and synthesising literature evidence, we analyse the mechanisms of retrieval failure and summarise advanced technical solutions, aiming to optimise success rates for challenging retrievals and mitigate risks associated with long-term filter retention.

## 2 Case presentation

A 73-year-old male presented with acute bilateral lower limb swelling for 1 day, 6 years after implantation of an IVCF (Günther Tulip, COOK). The patient reported a sudden onset of lower limb heaviness and weakness upon waking. His medical history revealed a prior diagnosis of bilateral lower extremity DVT with PE 6 years earlier. In order to prevent thrombus detachment from aggravating the symptoms of PE, he underwent IVCF placement and pulmonary artery thrombolysis therapy. Postoperative anticoagulation therapy resulted in clinical improvement, but a 1-month follow-up revealed residual thrombus in the lower extremity and pulmonary arteries, prompting deferred filter retrieval. He was advised to return for reevaluation in 1–2 months, but instead sought care at a local clinic, where persistent thrombosis led to a recommendation for indefinite anticoagulation (rivaroxaban 20 mg daily). The patient subsequently discontinued anticoagulation 1 month before admission due to concerns about perioperative bleeding risk during urolithiasis surgery.

**Figure 1:**
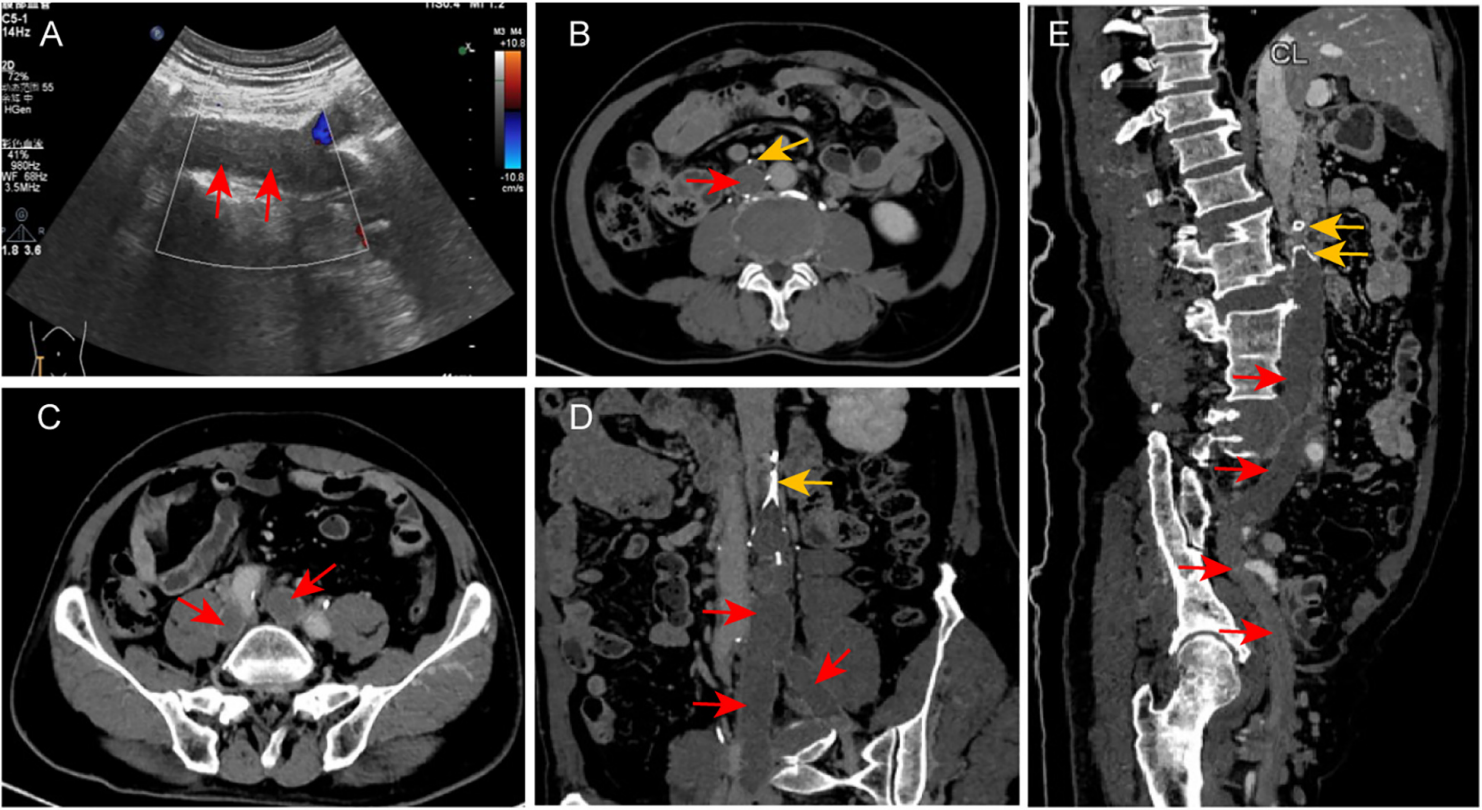
Imaging findings on admission. **(A)** Abdominal vascular ultrasound demonstrates hypoechoic thrombus (red arrows) in the IVC and bilateral iliac veins with absent Doppler flow signals, consistent with thrombosis. **(B–E)** CTV reveals: The implantation status of the patient's IVCF. The filter head and the retrieval hook are attached to the wall. There are extensive low-density shadows in the IVC below the filter, and the bilateral common iliac and external iliac veins, indicating extensive thrombosis (the red arrow points to the thrombus, and the yellow arrow points to the filter and its leg branches).

Physical examination: The patient's thighs were obviously swollen, but the skin colour was normal, and no ecchymosis was observed. Due to the swelling, the skin tension was high. The skin temperature was normal, and there was no obvious tenderness. No obvious abnormalities were found in the arteries of both lower limbs. Laboratory tests: Haemoglobin: 146 g/L (110–160 g/L); WBC 10.04 × 10⁹/L (4.0–10.0 × 10⁹/L), D-dimer >20 μg/ml (<0.5 μg/ml), FDP 66.43 μg/ml (<5 μg/ml). No abnormalities were found in hepatic and renal function. Ultrasound of the lower limb vessels showed: blood stasis in the deep veins of both lower limbs, with no obvious signs of thrombosis in the femoral and popliteal veins. Thrombosis of the left peroneal vein and the anterior branch of the posterior tibial vein, and thrombosis of the right calf intermuscular vein. Abdominal vascular ultrasound: Thrombosis of the IVC and bilateral common iliac veins (Figure 1A). CT venography (CTV): The filter and the retrieval hook were attached to the vessel wall, and the distal leg branch had penetrated the vessel wall (Figures 1B,C). Extensive thrombosis in the infra-filter IVC, bilateral common iliac veins, and external iliac veins (Figures 1B−E).

**Figure 2:**
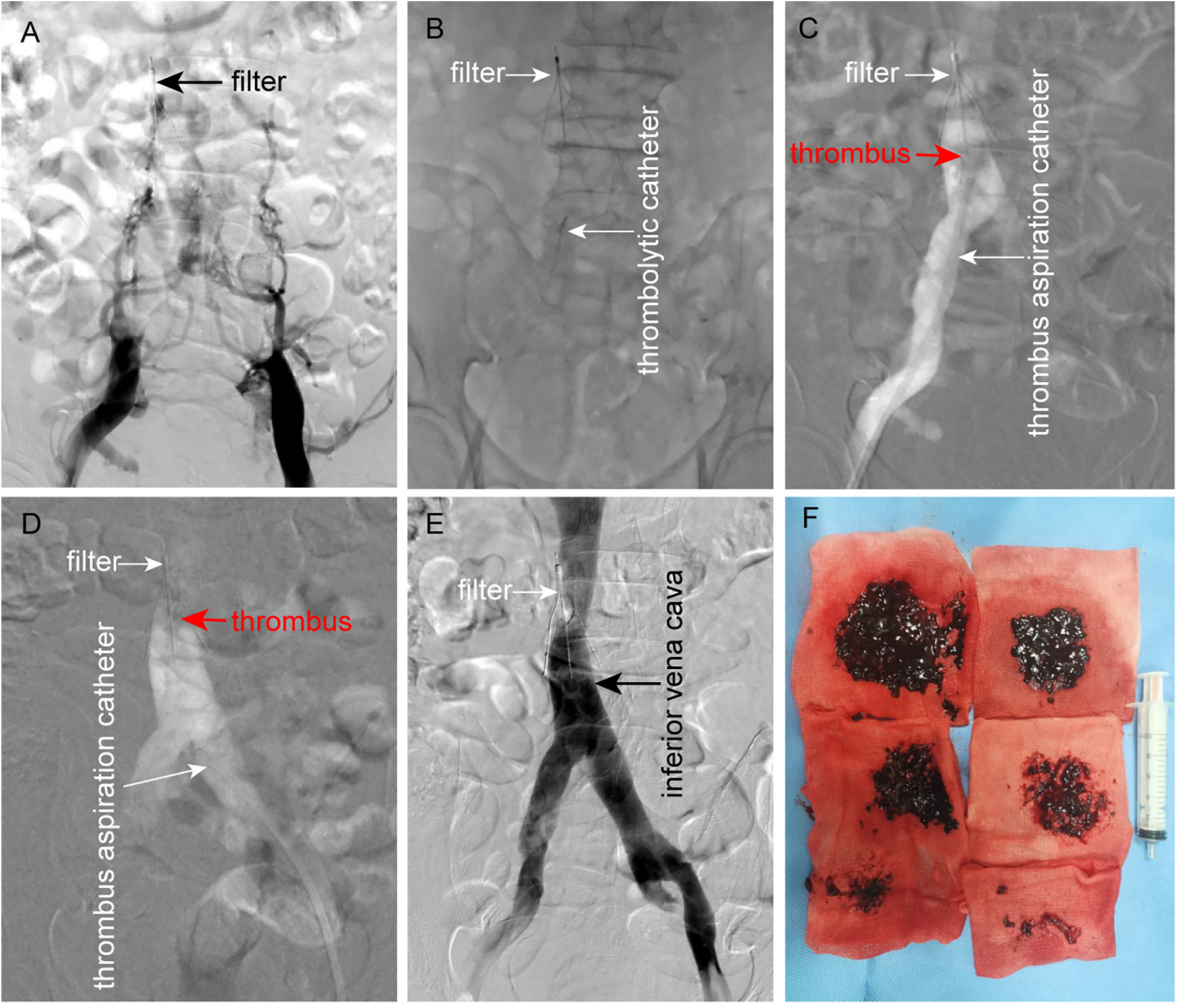
Procedural steps of CDT and mechanical thrombectomy. **(A)** DSA demonstrates complete filling defects in the bilateral iliac veins and IVC, with collateral drainage via lumbar veins and extensive paravertebral collaterals. **(B)** Antegrade pulsed-spray infusion of 400,000 IU urokinase through a 4F thrombolytic catheter traversing the thrombus from distal to proximal. **(C, D)** Mechanical thrombectomy via bilateral femoral venous access using a 10F thrombus aspiration catheter, achieving near-complete clearance of iliac and IVC thrombi. **(E)** Post-procedural DSA confirms restored patency of the iliac veins and IVC, with minimal residual thrombus adherent to the filter. **(F)** Extracted thrombi comprising a large amount of fresh thrombus and a small amount of old thrombus.

Following admission, anticoagulation therapy was promptly initiated with enoxaparin sodium injection (100 IU/kg, subcutaneous injection, every 12 h). Due to the extensive thrombotic burden, the patient underwent catheter-directed thrombolysis (CDT) and mechanical thrombectomy under local anesthesia after multidisciplinary consultation and informed consent. Initial digital subtraction angiography (DSA) revealed complete occlusion of the bilateral iliac veins and entire IVC, with compensatory filling via lumbar veins and extensive collateral circulation (Figure 2A). After mapping the thrombus distribution, pulsed-spray thrombolysis was performed using a 4F catheter to deliver 400,000 IU urokinase sequentially from distal to proximal within the thrombus (Figure 2B). Ten minutes later, mechanical thrombectomy was conducted using a 10F thrombus aspiration catheter (AcoStream, China), achieving near-complete clearance of thrombi from the IVC and iliac veins (Figures 2C,D). After multiple aspirations, except for a small amount of thrombus firmly attached to the filter that could not be removed, the thrombus in the IVC and iliac vein was almost completely removed. Most of the thrombi extracted were soft fresh thrombi, accompanied by a small amount of tough old thrombi (Figures 2E,F).

**Figure 3:**
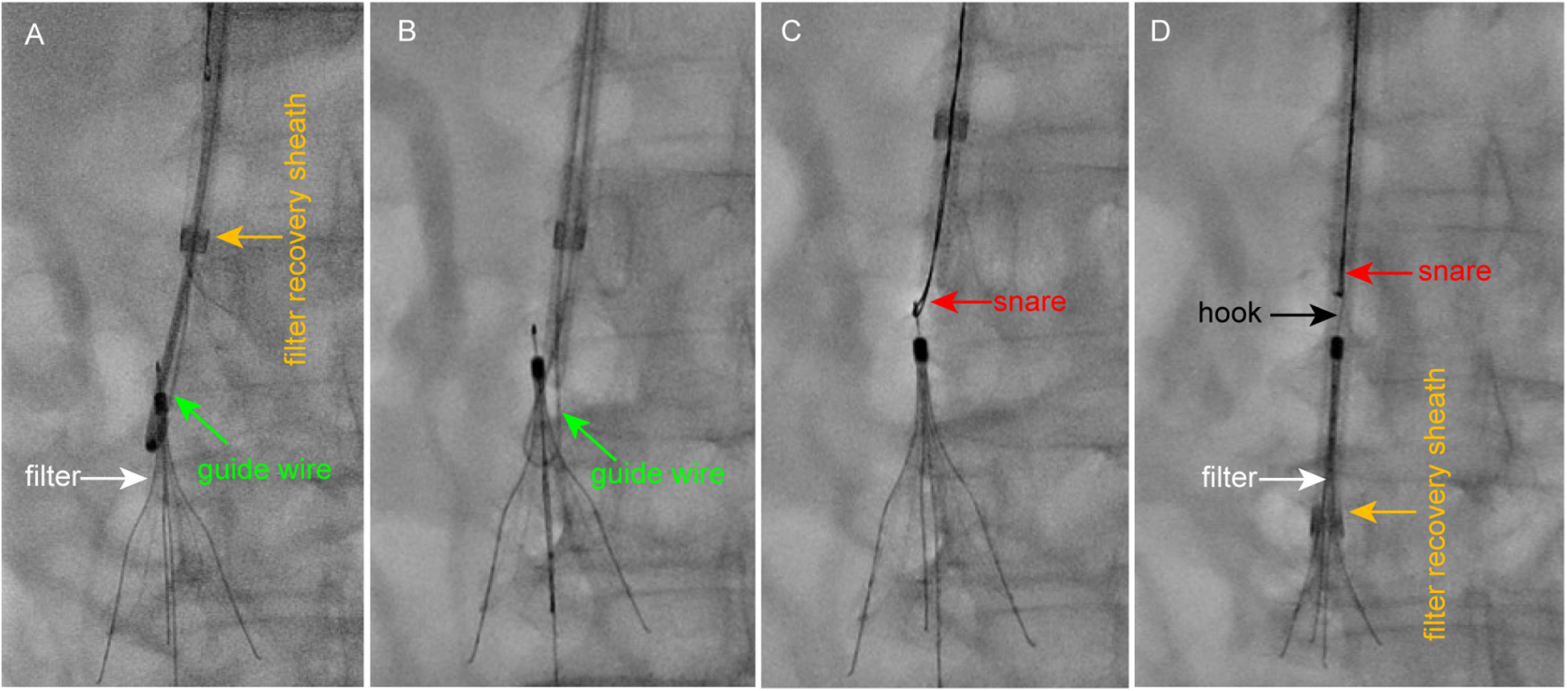
Procedural steps of loop snare technique for filter retrieval. **(A)** A guidewire is looped around the filter neck with assistance from a pigtail catheter, and its tip is externalized using a snare device. **(B)** The looped guidewire dissects fibrotic tissue to free the retrieval hook from the adherent caval wall. **(C)** The snare successfully engages the freed retrieval hook. **(D)** Despite straightening the hook, the filter remains firmly anchored by perforated struts and cannot be fully retrieved.

Following near-complete thrombus clearance and considering the risks associated with prolonged filter retention, retrieval was attempted after obtaining informed consent from the patient and his relative. However, standard retrieval methods failed due to wall apposition and dense fibrotic adhesion between the filter and the caval wall. A loop snare technique was subsequently employed: a loop-forming guidewire was used to dissect fibrotic tissue encapsulating the retrieval hook, which was then successfully engaged by a snare (Figures 3A–C). Nevertheless, the filter remained firmly anchored to the IVC by perforated distal struts enmeshed in fibrotic tissue. Even after straightening the retrieval hook, the filter could not be withdrawn into the retrieval sheath due to persistent adhesion (Figure 3D).

**Figure 4:**
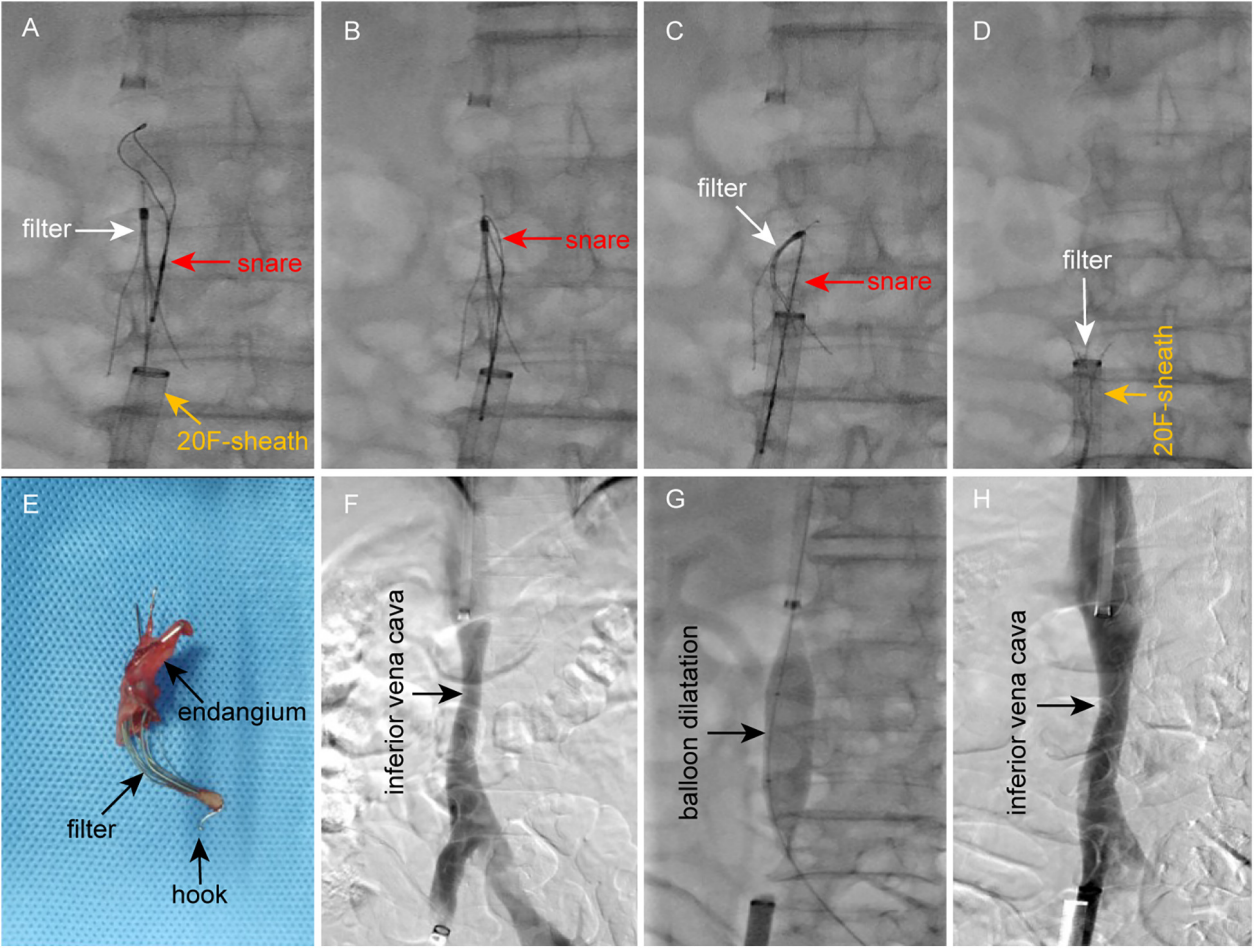
Filter retrieval and IVC balloon angioplasty. **(A–D)** Retrograde retrieval of the IVC filter via femoral venous access using a 20F vascular sheath. **(E)** Retrieved filter demonstrating deformation but structural integrity. **(F)** Post-retrieval DSA confirms IVC patency without contrast extravasation, with mild stenosis at the filter implantation site. **(G)** Balloon angioplasty for IVC stenosis. **(H)** Post-angioplasty DSA shows significant luminal improvement.

Given the straightened retrieval hook and the persistent anchoring by embedded struts, re-engagement from a superior approach was deemed unfeasible. After analysing the biomechanical constraints, a retrograde femoral approach was pursued. A 20F vascular sheath (Gore, USA) was deployed, and the straightened hook was recaptured inferiorly using a snare device. The filter was then retracted into the large-bore sheath under x-ray fluoroscopic guidance (Figures 4A–D). Despite deformation and entanglement of struts with neointimal tissue, the filter was extracted intact without fracture or residual fragments (Figure 4E). The patient reported only mild, transient lumbar discomfort during retrieval, with no other adverse symptoms. Post-procedural DSA confirmed no evidence of vascular rupture or haemorrhage. Mild IVC stenosis secondary to chronic filter incorporation was treated with 16-mm balloon angioplasty, achieving significant luminal improvement (Figures 4F–H).

Postoperatively, the patient continued subcutaneous enoxaparin sodium injection for anticoagulation, supplemented with *Zhilong Huoxue Tongyu Granules* (a traditional Chinese herbal formula for promoting blood circulation) and oral *Aescuven Forte* (300 mg twice daily) to enhance venous tone and reduce edema. By postoperative day 2, the lower limb swelling had markedly subsided. Repeat Doppler ultrasound confirmed recanalization of the IVC and iliac veins, with D-dimer levels decreasing to 0.79 μg/ml and FDP to 3.55 μg/ml. The patient was discharged on postoperative day 3 and transitioned to rivaroxaban (20 mg once daily) for continued anticoagulation. At the 3-month follow-up, the patient exhibited normal gait without edema. Follow-up ultrasound demonstrated no residual thrombosis in the lower extremities or abdominal vasculature, prompting discontinuation of anticoagulation therapy.

## 3 Discussion

The IVCF plays a critical role in preventing fatal PE (11). In recent years, the use of retrievable IVCF has increased significantly due to heightened awareness of venous thromboembolism (VTE), particularly PE. By 2012, an estimated 300,000 filters were implanted annually in the United States alone (12). Despite the predominant use of retrievable filters, retrieval rates remain suboptimal, averaging only 20%–30% (13, 14). This discrepancy implies that a substantial number of filters are retained indefinitely, substantially elevating the risk of filter-related complications (15). Long-term IVCF retention is associated with severe sequelae, including caval wall penetration, filter migration, fracture, IVC thrombosis, and recurrent DVT (6, 8, 12, 16, 17). The reported incidence of IVC thrombosis post-filter placement ranges from 2% to 30% (18, 19). Once the filter occlusion can lead to acute symptoms until phlegmasia alba dolens and whit probably progression. Therefore, thrombosis within the IVC often coexists with lower extremity DVT, leading to acute symptoms and, if untreated, progression to post-thrombotic syndrome (PTS). In this case, prolonged filter retention, coupled with trapped emboli and recent anticoagulation discontinuation, precipitated acute thrombosis in the IVC and iliac veins, exacerbating venous stasis.

To standardise IVCF use and mitigate complications, the U.S. FDA issued a safety alert in 2010 recommending prompt removal when PE risk subsides, anticoagulation is feasible, and thrombus burden is minimal (<25% on venography) (20). Subsequent 2016 ACCP guidelines reinforced this stance, advocating filter retrieval immediately after PE risk resolution (1, 21). Additionally, risk-benefit analyses suggest optimal retrieval within 29–54 days post-implantation (22). Therefore, after the filter is implanted, patients should undergo close follow-up, particularly during the retrieval window, to assess thrombus resolution and facilitate timely planning for safe filter removal. While standard techniques suffice for most retrievals within this retrieval time window, complex cases, such as severe filter tilt (>15°), wall apposition, or endothelialisation, often necessitate some advanced techniques. Our clinical experience corroborates that adherence to the retrieval time window significantly improves success rates. Conversely, retrieving chronically retained filters (e.g.,>3 years) poses formidable challenges, as standard tools (e.g., snares and sheaths) frequently fail due to fibrotic adhesion and structural incorporation (15). Filter tilt, occurring in 3%–9% of cases, disrupts laminar flow, reduces shear stress, and triggers neointimal hyperplasia, thereby anchoring the filter to the caval wall (5). Retrieval hooks embedded in hyperplastic tissue further complicate engagement.

Failed retrievals demand advanced techniques beyond standard tools. However, these methods carry inherent risks. A recent systematic review and meta-analysis associated advanced retrieval with higher adverse event rates (23). Nonetheless, when performed by experienced operators, absolute complication rates remain low, and the long-term risks of filter retention often justify intervention. As summarised in Table 1, numerous advanced strategies, including loop snares, balloon angioplasty, laser-assisted extraction, and forceps dissection et al., have expanded retrieval success for refractory cases (9, 10). Despite these innovations, a subset of filters remains irretrievable due to extreme endothelialisation or patient-specific anatomical constraints.

**Table 1:** Advanced techniques for retrieval IVCF and their characteristics.

Advanced technique	Indications	Advantages	Possible complications	Ref.
Loop-snare technique (Sling Technique)	The filter tilted or apex embedded	No additional consumables required	No reports yet	(24–26)
Fall-back technique	The filter retrieval hook cannot be snared	High success rate and simple operation	No reports yet	(27)
Stiff wire-displacement technique	Filter tilted and apex adjacent to the cava wall	Displacing the filter apex toward the cava lumen	Filter element migration, deformation and breakage	(8, 28)
Balloon-displacement technique	Filter tilted and apex embedded	The filter can be shimmied away from the cava	No reports yet	(8, 29)
Dual-access technique	Single-access approach fails to displace the tilted filter	Release the filter apex from the cava	Pelvic vein lacerations	(10)
Sandwich technique	The filter recalcitrant	It can be freed or untilted	Blood vessel wall injury	(30)
Forceps technique	Filter adhesion and the hook embedded	Expose the filter fully and clamp it	Blood vessel wall injury	(24, 31, 32)
Laser sheath technique	Filter with a longer indwelling time	Dissect fibrous tissue and release adhesions	Blood vessel wall injury	(33)
Double vascular sheaths docking technology	Dislocation of the filter	Minimally invasive and efficient	No reports yet	(34)

Currently, endovascular techniques remain the primary approach for IVCF removal. However, when filters are irretrievable due to severe complications or refractory to minimally invasive interventions, surgical options (laparoscopic or open retrieval) may be considered (35, 36). Laparoscopic retrieval is primarily suitable for filters with anteriorly displaced tips protruding outside the IVC (37). Conversely, filters with posteriorly embedded tips or deep caval wall incorporation pose significant challenges for laparoscopic approaches. Although open surgical retrieval is invasive, it remains a viable salvage option when endovascular and laparoscopic methods fail or result in complications (35, 38).

In this case, the filter had been retained for over 6 years, complicated by IVC and iliac vein thrombosis, wall apposition, fibrotic adhesion, and strut perforation. Management was particularly challenging. First, thrombus clearance was prioritised to restore venous outflow and alleviate lower limb edema. Drawing on prior experience in IVC thrombosis management (39), we employed CDT combined with mechanical thrombectomy using an aspiration catheter. Fortunately, near-complete thrombus clearance created optimal conditions for subsequent filter retrieval. During the filter removal process, as expected, this filter was extremely stubborn, and we tried several techniques, but none of them worked. Even straightening the filter retrieval hook failed to pull it out. Analysis of the filter's biomechanical integration revealed extensive endothelial overgrowth, anchoring the device and perforated struts enmeshed in fibrotic tissue. Excessive force risked catastrophic vascular injury. Consequently, we changed our strategy and used a larger vascular sheath (20F) to remove it retrogradely from the femoral vein. The large-bore sheath accommodated the deformed filter, while altering the traction vector allowed disengagement from the caval wall. Despite minor neointimal detachment, the procedure concluded without hemodynamic instability or significant pain. Post-procedural angiography confirmed no vascular injury. This case demonstrates that for chronically retained, adherent IVCF, particularly conical designs like the Günther Tulip, retrograde retrieval via large-bore femoral access is a safe and feasible alternative when jugular approaches fail. This strategy expands the armamentarium for refractory filter extraction, emphasising biomechanical optimisation over brute force.

## 4 Conclusion

Prolonged retention of IVCF carries significant risks of filter-related complications, including life-threatening thrombosis and vascular injury. Strict adherence to implantation criteria and timely retrieval within the recommended window are paramount to mitigate these risks. In cases complicated by IVC thrombosis, prompt thrombus clearance is essential to establish venous patency and enable safe filter retrieval. While converting a retrievable filter to a permanent device may be justified in critically ill or high-risk patients with limited procedural tolerance, aggressive retrieval using advanced techniques should be pursued whenever clinically feasible. For stubborn conical filters (e.g., Günther Tulip), retrograde retrieval via a large-bore sheath offers a safe and effective remedial strategy when standard jugular approaches fail. This technique capitalises on the sheath's capacity to accommodate deformed filters while minimising radial traction forces. Crucially, advanced retrieval procedures demand execution by experienced interventionalists to optimise success rates and minimise adverse events.

## Data Availability

The original contributions presented in the study are included in the article/Supplementary Material, further inquiries can be directed to the corresponding author.
